# Determining the optimal stage for cryopreservation of human embryonic stem cell-derived retinal pigment epithelial cells

**DOI:** 10.1186/s13287-022-03141-2

**Published:** 2022-09-05

**Authors:** Ting Zhang, Xianyu Huang, Sujun Liu, Xinyue Bai, Xinyue Zhu, Dennis O. Clegg, Mei Jiang, Xiaodong Sun

**Affiliations:** 1 National Clinical Research Center for Ophthalmic Diseases, Shanghai, China; 2 Department of Ophthalmology, Shanghai General Hospital (Shanghai First People’s Hospital), Shanghai Jiao Tong University, School of Medicine, 100 Haining Road, Shanghai, 200080 China; 3 National Clinical Research Center for Eye Diseases, Shanghai, China; 4 Shanghai Key Laboratory of Fundus Diseases, Shanghai, China; 5 Department of Molecular, Cellular and Developmental Biology, University of California, Santa Barbara, USA; 6 Shanghai Engineering Center for Visual Science and Photomedicine, Shanghai, China

**Keywords:** Retinal pigment epithelium, Cryopreservation, Cell cycle, Extracellular matrix, THBS1

## Abstract

**Background:**

Human embryonic stem cell-derived retinal pigment epithelial cells (hESC-derived RPE) are a promising source for cell-replacement therapy to treat retinal degenerative diseases, but research on RPE cryopreservation is limited. This study aimed to determine the best phase for RPE cryopreservation to preserve the post-thaw function and uncover the mechanism underlying RPE freezing tolerance.

**Methods:**

hESC-derived RPE cells were cryopreserved at various time points after seeding. After thawing, the survival and attachment rates, RPE marker gene expression, apical-basal polarity, PEDF secretion, transepithelial resistance, and phagocytotic ability of post-thaw RPE cells were evaluated. RNA sequencing was performed on RPE cells at three-time points, differentially expressed genes were identified, and gene ontology, Kyoto encyclopedia of genes and genomes, and protein–protein interaction analyses were used to investigate the key pathways or molecules associated with RPE cell freezing tolerance.

**Results:**

RPE frozen at passage 2 day 5 (P2D5) had the highest cell viability and attachment after thawing. They also retained properly localized expression of RPE marker genes and biological functions such as PEDF secretion, high transepithelial resistance, and phagocytic ability. The RNA-sequencing analysis revealed that RPE cells at P2D5 expressed high levels of cell cycle/DNA replication and ECM binding associated genes, as well as THBS1, which may serve as a possible hub gene involved in freezing tolerance. We also confirmed that the RPE cells at P2D5 were in the exponential stage with active DNA replication.

**Conclusions:**

We propose that freezing hESC-derived RPE cells during their exponential phase results in the best post-thawing outcome in terms of cell viability and preservation of RPE cell properties and functions. The high expression levels of the cell cycle and ECM binding associated genes, particularly THBS1, may contribute to better cell recovery at this stage.

**Supplementary Information:**

The online version contains supplementary material available at 10.1186/s13287-022-03141-2.

## Background

The retinal pigment epithelium (RPE) is a pigmented cell monolayer between the neural retina and the choroid. It is required for the maintenance of the blood-retinal barrier (RBB), the secretion of growth factors, the recycling of retinoids, the phagocytosis of photoreceptor outer segments (OS), the delivery of nutrients, and the disposal of metabolic waste products [1]. The occurrence and progression of many retinal degenerative diseases, such as age-related macular degeneration (AMD), retinitis pigmentosa (RP), and Stargardt's disease, are closely linked to RPE dysfunction and death, which eventually leads to the degeneration of overlying photoreceptor cells [2]. As a result, RPE cells derived from human embryonic stem cells (hESC) could provide an unlimited source of RPE replenishment and better support for the overlying photoreceptor cells [3, 4]. The safety and efficacy of cell-replacement therapy as a treatment for retinal degenerative diseases are being tested in phase I/II clinical trials using hESC-derived RPE [5–8] (for more information, visit http://www.clinicaltrials.gov). To date, much emphasis has been placed on developing methods to differentiate hESCs to RPE, the best delivery strategy, and the development of scaffolds for RPE transplantation [9–13]. However, research on RPE cryopreservation to preserve post-thaw function is still limited.

Cryopreservation is the most widely used method of storing biomaterials, which can be deep-frozen and stored in liquid nitrogen for an almost infinite time. The differentiation of human pluripotent stem cells toward RPE is laborious and time-consuming, and once obtained, RPE cells can only proliferate for a limited number of passages before the cells undergo an epithelial-to-mesenchymal transition (EMT). As a result, efficient cryopreservation of hESC-derived cells is required for clinical use to maximize shelf life and ensure convenient transportation for later on-demand distribution [14]. Successful cryopreservation can result in high cell recovery and viability, as well as the preservation of cell function after the freeze–thaw cycle. However, the importance of selecting the optimal stage/phase for RPE cryopreservation has always been underestimated in optimizing freezing medium and cooling strategies [15–17]. Some studies suggested that RPE cells be frozen shortly after passaging when they have not reached confluency or regained pigment [18, 19]. Other reports used other time points for cryopreservation [20–23]. It is still unclear whether the phase/stage of cell culture used for cryopreservation affects post-thaw RPE functions. Furthermore, the mechanism underlying RPE cell tolerance to freezing has not been investigated. Identifying key molecules or pathways involved in this process could aid in optimizing cryopreservation strategies.

In this study, we cryopreserved hESC-derived RPE cells at various time points after seeding and evaluated the characteristics and functions of post-thaw RPE cells. We discovered that cryopreservation of RPE at day 5 after passaging (P2D5) resulted in a better post-thaw outcomes than other time points, as evidenced by higher cell survival and attachment rate, higher expression of RPE markers, polarized cell morphology, increased PEDF secretion, higher transepithelial resistance, and improved phagocytic function. The RNA-seq analysis revealed that RPE cells at P2D5 showed higher expression of genes associated with cell cycle/DNA replication and extracellular matrix (ECM) binding, which could explain the freezing tolerance of cells at this stage. We also confirmed that the hESC-derived RPE cells were in the exponential phase with active DNA replication at P2D5. Overall, our findings show that the exponential phase of cell growth (P2D5) is the best stage for hESC-derived RPE cryopreservation.

## Methods

### Cell culture

Human embryonic stem cells (hESC) (H9, Wicell, USA) were differentiated using a 14-day differentiation protocol by sequential exposure to various molecules including Nicotinamide, Noggin, Activin A and IGF-1, as previously described [18]. The induced cells were then dissociated with TryPLE (Thermo Fisher Scientific) and cultured in XVIVO-10 for another 4–5 weeks on matrigel-coated surface with the seeding density of 10^5^ cells/cm^2^. During this period, the cells started to exhibit the characteristic hexagonal morphology with pigmentation, which were defined as Passage 0 (P0). The RPE cells were further cultured in XVIVO-10 for 4–5 weeks before they were subjected to the subsequent passages or characterization except phagocytosis assay, for which the cells were cultured in “Miller” medium (MEM alpha supplemented with 5% FBS, 1% N1 supplement, NEAA (1X), Glutamax (1X), 250 μg/ml taurine, 20 ng/ml hydrocortisone, 0.013 ng/ml triiodo-thyronin) [24]. To generate polarized RPE monolayers, cells were seeded at 10^5^ cells/cm^2^ onto matrigel-coated transwell membranes (0.4 μm pore, Corning). Media were refreshed twice a week.

### Cryopreservation and thawing experiments

For cryopreservation experiments, the RPE cells at various time points were dissociated with TryPLE and frozen in CryoStor CS10 (Biolife Solutions) or Genxin (Selcell) at a concentration of 2 × 10^6^ cells/ml. Cells were immediately placed in a Nalgene Cryo freezing container, frozen at − 80 °C overnight to achieve a cooling rate of − 1 °C/min, and then transferred to liquid nitrogen for long-term storage. Rapid thawing was carried out using ThawSTAR CF2 Automated Thawing System (BioLife Solutions) and cells were diluted by dripping pre-warmed XVIVO-10 medium and centrifuged at 250×*g* for 3 min. After resuspension in XVIVO-10 medium, the thawed RPE cells were counted with a hemocytometer to determine the recovery rate and survival rate using standard trypan blue exclusion (trypan blue stain 0.4%, Thermo Fisher). Cells were then cultured on matrigel-coated surfaces at 105 viable cell/ml in XVIVO-10 with the addition of Y27632 (final concentration: 10 μM), which was withdrawn 24 h later. The attachment rate was determined 24 h after thawing by dissociating the cells again with TryPLE and counting the cell number.

### Analysis of cell growth

hESC-derived RPE cells at P1D35 were passaged and seeded on multiple matrigel-coated wells in a 12-well plate at a density of 10^5^/cm^2^. At the indicated time points during the culture period, the RPE cells were harvested by dissociation with TryPLE and resuspended in 1 ml culture medium. Cell number was then determined by counting with a hemocytometer.

### EdU labeling assay

EdU is an efficient and convenient method to detect proliferating cells both in vitro and in vivo. The EdU labeling of P2 RPE was carried out at different time points during culture and was detected using Click-iT EdU Imaging Kits (Thermo Fisher Scientific) following the manufacturer’s protocol. Briefly, hESC-derived RPE cells at P1D35 were passaged and seeded on multiple matrigel-coated coverslips at a density of 10^5^/cm^2^. At indicated time points, the RPE cells were incubated with 10 μM EdU in the medium for 24 h, and were then fixed, permeabilized and stained with the Click-iT reaction cocktail for the detection of incorporated EdU. Images from 5 random fields were captured (Axio Observer A1, Zeiss), and the percentages of EdU-positive cells were calculated.

### Immunofluorescence staining

Immunofluorescence staining of hESC-derived RPE cells was performed as previously described [25]. Briefly, the RPE cells were fixed with 4% paraformaldehyde for 15 min, permeabilized with 50% ethanol for 5 min, and blocked with a solution containing 5% normal goat serum, 0.5% BSA and 0.05% saponin for 30 min. The cells were then incubated overnight at 4 °C with primary antibodies that were diluted in the blocking solution. After several washes with PBS, cells were incubated with Alexa Fluro-conjugated goat anti-rabbit secondary antibodies (Thermo Fisher Scientific) for 1 h at 25 °C. Cells were then stained with Phalloidin-647 (1:1000, Abcam) to visualize the actin cytoskeleton and subsequently DAPI to counterstain nuclei. The following primary antibodies were used: anti-RLBP1 (1:200; Proteintech 15356-1-AP), anti-ZO-1 (1:200; Thermo Fisher Scientific 33–9100), and anti-BEST1 (1:1000; Abcam ab2182). The images were captured with Leica TCS SP8 confocal laser scanning microscope. To observe polarized properties of the RPE cultured on transwell membranes, Z-stack confocal microscopy was performed. For quantification of immunofluorescence staining, 5 fields were randomly chosen for each experiment.

### Quantitative real-time PCR (qPCR)

Total RNA from RPE cells was extracted using the RNAsimple Total RNA Kit (Tiangen), and the cDNA was synthesized using PrimeScript RT Master Mix (TaKaRa). TB Green Fast qPCR Mix (Takara) and the ViiA7 real-Time PCR System (Thermo Fisher) were used to amplify cDNA with a program consisting of 40 cycles of amplification (T_m_ = 60 °C). Gene-specific primers were synthesized by Shanghai Sunny Biotechnology and the sequences of the primers are listed in Table S1. GAPDH gene expression was used as an internal control. mRNA levels of target genes were calculated using the 2^−ΔΔCt^ method. A minimum of three biological replicates from two independent experiments were analyzed.

### Phagocytosis assay

Porcine rod outer segments (POS) were isolated from fresh porcine eyes, labeled with Alexa Fluor 488 NHS Ester (Succinimidyl Ester, Thermo Fisher) and aliquoted as previously described [26]. Phagocytosis assay was performed on the RPE cells cultured in a 96-well plate following a previously described protocol [27]. Briefly, the hESC-derived RPE cells were seeded in quadruplicate in a matrigel-coated 96-well plate at a concentration of 10^5^/well and allowed to grow for 4–5 weeks in “Miller” medium. On the day of phagocytosis assay, the thawed ROS aliquots were centrifuged at 2400×*g* for 5 min and resuspended in “Miller” medium in the concentration of 10^7^ POS/ml. 100 µl of ROS per well was applied to the RPE cells for 3.5 h at 37 °C. The unbound ROS were washed away by rinsing with DPBS containing Ca^2+^ and Mg^2+^ and the RPE cells were immediately fixed with 4% paraformaldehyde in DPBS. The plate was then scanned with Tecan Spark microplate reader (Tecan, Austria) for the quantification of the fluorescence signal. To control for different cell numbers in different wells, cells were stained with Hoechst 33342 and the fluorescence intensity was also determined by the microplate reader. The phagocytic ability of RPE cells was therefore represented as the value of POS fluorescence intensity/ Hoechst fluorescence intensity.

### Measurement of TEER (transepithelial electrical resistance)

For the measurement of TEER, the RPE cells were grown on matrigel-coated transwell membranes. A membrane without cells was used as a background control. After 4–5 weeks of culture, TEER of the RPE cells was measured using an epithelial voltmeter (World Precision Instruments) and calculated by subtraction of background TEER from the control membrane. The TEER per unit area (Ω.cm^2^) was obtained by multiplying the raw TEER value by the surface area of the transwell membrane (0.33 cm^2^).

### Enzyme-linked immunosorbent assay (ELISA)

To determine secretion of PEDF, hESC-derived RPE cells were thawed and cultured onto matrigel-coated transwell membranes. At Day 35 post thaw, the apical- and basal- side conditioned media were collected after 24 h of culture and the concentration of PEDF protein was measured by ELISA following the manufacturer’s instructions (BioVendor Laboratory Medicine).

### RNA-seq library construction

Three independent batches of hESC-derived RPE cells at P1D35, P2D5 and P2D11 (9 samples in total) were subjected to RNA sequencing. Total RNA was extracted with Trizol (Thermo Fisher), and mRNA was purified by using poly-T oligo-attached magnetic beads. The RNA was reversed transcribed by using random hexamer primer and M-MuLV Reverse Transcriptase (NEB). The double-stranded cDNA was amplified using KAPA HiFi HotStart ReadyMix (KAPA Biosystems) and purified using AMPure XP beads (Bechman Coulter). After quantification by Qubit2.0 Fluorometer, cDNA was applied to a Bioanalyzer 2100 on a High-Sensitivity DNA Chip (Agilent Bioanalyzer) to check the library size distribution. All sample libraries were sequenced on an Illumina NovaSeq 6000 to generate 150 bp paired-end reads (Novogene, Beijing).

### RNA-seq data analysis

Clean data were obtained from FastQ raw data by removing adapter, ploy-N sequences and low-quality reads. All the downstream analyses were based on the clean data with high quality. The reads were mapped to the hg38 version of human genome using TopHat2 version 2.0.9 program [28]. We calculated fragment per kilobase per million (FPKM) as expression level using the FeatureCounts (v1.5.0-p3) [29]. Genes with the FPKM > 1.0 in at least one sample across all samples were retained for further analysis, and the expression levels were transformed to log-space by using the log2 (FPKM + 1). Differentially expressed genes (DEGs) among different samples were identified using DESeq2 R package (1.20.0) [30]. The resulting P-values were adjusted using the Benjamini and Hochberg approach for controlling the false discovery rate (FDR). Genes with *P*_adj_ values < 0.05 and log2 fold change > 1 were assigned as differentially expressed genes. DEG heat maps were clustered by hierarchical clustering and visualized using Java Tree View software [31]. Principal component analsysis (PCA) analysis was performed using R (http://www.r-project.org). Gene Ontology (GO) enrichment analysis of differentially expressed genes was implemented by the clusterProfiler R package (3.8.1), in which gene length bias was corrected [32]. GO terms with corrected P value < 0.05 were considered significantly enriched by differential expressed genes. The KEGG database was used to analyze high-level cellular functions (http://www.genome.jp/kegg) [33]. We used clusterProfiler R package (3.8.1) to test the statistical enrichment of differential expression genes in KEGG pathways. Then, a protein–protein interaction network of KEGG enriched DEGs was analyzed using the Search tool for the retrieval of interacting genes (STRING database, http://string-db.org/) [34]. An interaction with a combined score higher than 0.4, which is a widely used threshold, was considered statistically significant.

### Statistical analysis

All statistical analyses were performed in GraphPad Prism software (GraphPad Prism 8), and data were presented as mean ± SD. Student's *t* test (two-tailed) was performed for statistical analysis between two groups. One- or two-way ANOVA with Tukey’s or Bonferroni's multiple comparison post hoc test was used when three or more groups were compared. The statistical analyses were obtained from three biological replicates or independent experiments. Statistical significance was set at *P < 0.05.

## Results

### Frozen RPE cells at P2D5 achieved the highest cell viability and attachment after thawing

**Fig. 1:**
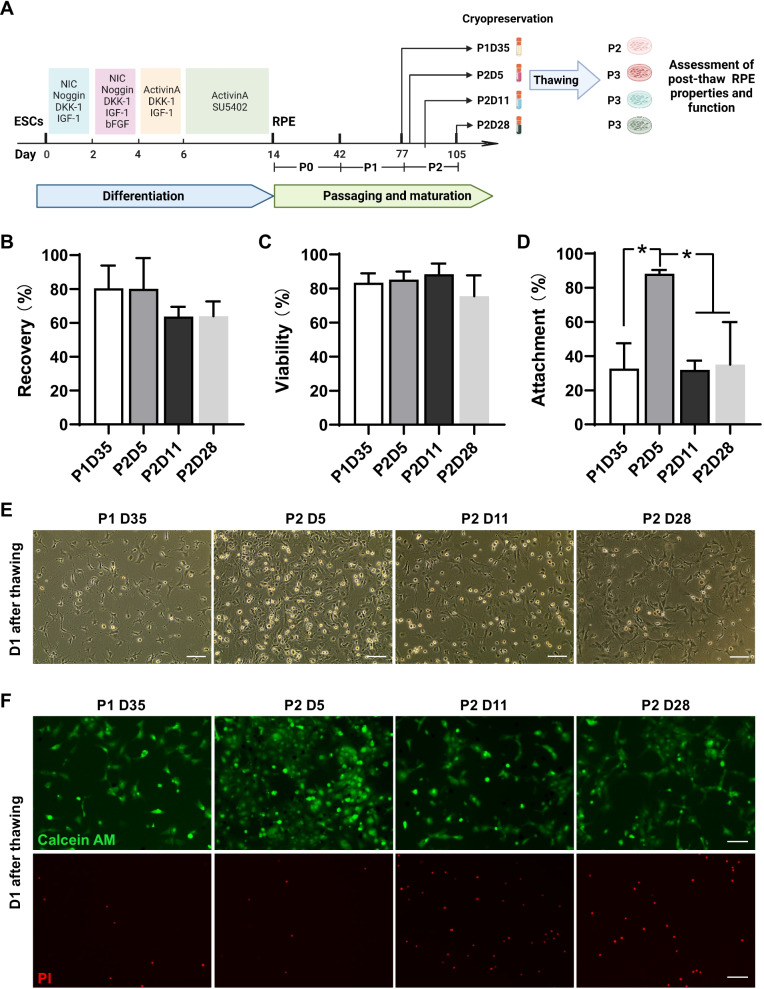
hESC-derived RPE cells frozen at P2D5 achieved the highest cell viability and attachment after thawing. **A** The flow diagram for hESC-derived RPE cell differentiation, passaging, and cryopreservation (created with BioRender.com). **B**, **C** The recovery rate (**B**) and viability rate (**C**) were measured upon thawing of hESC-derived RPE cells frozen at indicated time points. **D** The attachment rate was measured 24 h post thaw. *n* = 3 independent experiments; Data are represented as mean ± SD. Statistical differences are evaluated with one-way ANOVA with Tukey's post hoc test. *P < 0.05. **E** The bright-field images of RPE cells from different groups 24 h post thaw.**F** . At 24 h post thaw, the RPE cells were stained with Calcein AM/PI, where live and dead cells were depicted as green and red fluorescence, respectively. Scale bars: 100 μm

Consistent with previous studies [35], we observed that when RPE were dissociated and replated, the cells underwent a period during which they lost their classic hexagonal RPE morphology and pigmentation, and proliferated until they formed a monolayer again. This period usually lasted 10–14 days after passaging, then the cells readopted their hexagonal morphology and slowly gained pigmentation after around 4 weeks of culture. Accordingly, we selected four different timepoints P1D35, P2D5, P2D11 and P2D28 (Fig. 1A), to determine the optimal stage for cryopreservation of hESC-derived RPE cells. We started with hESC-derived RPE cells at passage 1 day 35 (P1D35). After passage, these cells were labeled as passage 2 (P2). We froze the cells in CryoStor CS10, a widely used commercial freezing solution, for 1–3 months before being subjected to thaw (Fig. 1A). After thawing, some cells were damaged and degenerated into subcellular debris, some cells underwent apoptosis, and the remaining cells survived. Hence, it is important to analyze the recovery and survival rates, apoptosis and attachment. The recovery rate was calculated as the live cell number/the number of cells initially frozen. The survival rate was calculated as the live cell number/the total cell number after thawing. The recovery rate for cells frozen at P1D35, P2D5, P2D11 and P2D28 was 80%, 80%, 64% and 64%, respectively, while their viability was 83%, 85%, 88% and 76%, respectively. The experiments were repeated three times. No significant recovery or survival rate differences were observed (Fig. 1B, C). At 24 h after thawing, cells were dissociated, and the cell number was counted. The attachment rate was then calculated as the attached cells/the seeded cells. RPE cells frozen at P2D5 showed a higher attachment rate than other time points (Fig. 1D, E). Cell death and viability were also assessed using Calcein-AM/PI staining at D1 post thaw. The RPE frozen at P2D5 had the highest viability (Fig. 1F), indicating that hESC-derived RPE cells at P2D5 perform better in the freeze–thaw cycle than other time points.

### Frozen RPE at P2D5 showed highly expressed and properly localized RPE markers after thawing

**Fig. 2:**
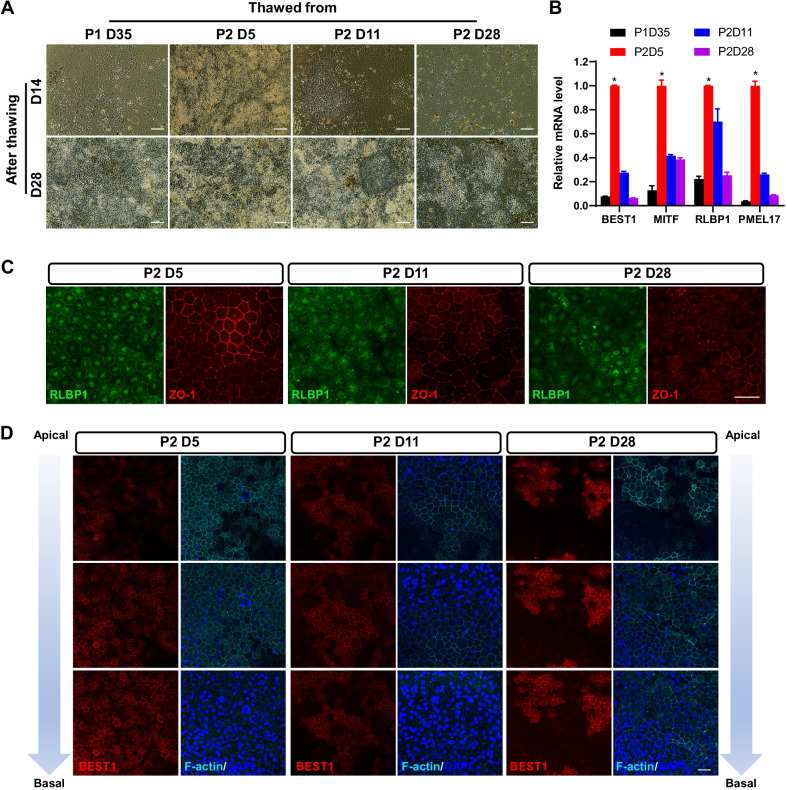
Frozen RPE at P2D5 showed highly expressed and properly localized RPE markers after thawing. **A** The bright-field images of thawed RPE cells frozen at indicated times at D14 (upper row) and D28 (lower row) post thaw, respectively. **B** RT-qPCR analysis of relative mRNA expression levels of RPE marker genes BEST1, MITF, RLBP1, and PMEL17 at D28 post thaw. GAPDH expression was used as internal control, and the values were normalized to the P2D5 group for each marker gene. *n* = 3 biological replicates. Data are represented as mean ± SD. Statistical differences are evaluated with two-way ANOVA with Bonferroni's post hoc test. *P < 0.05. **C** Immunostaining analysis for RLBP1 and ZO-1 expression in thawed RPE cells that were frozen at indicated time points at D28 post thaw, respectively. **D** Immunostaining analysis for BEST1 and F-actin (stained with Phalloidin) along the apical-to-basal axis of thawed RPE cells that were frozen at indicated time points at D28 post thaw, respectively. Nuclei were counter-stained with DAPI. Scale bars: 100 μm (**A**), 25 μm (**C**, **D**)

Although viability and attachment rates are important parameters for determining cell freezing success, the ultimate goal of freeze–thaw is to maintain normal RPE cell properties and function after thaw. RPE cells frozen at P2D5 displayed characteristic hexagonal morphology of RPE cells with distinct cell–cell adhesions (tight junctions) as early as 14 days after thawing, whereas cells frozen at other time points exhibited a fibroblastic phenotype, indicating they were experiencing an EMT transition for a relatively long period (Fig. 2A). RPE frozen at various time points had adopted typical RPE morphology by D28 post thaw, though heterogeneity was observed in the case of cells frozen at P2D11 and P2D28 (Fig. 2A). RT-qPCR at D28 post thaw revealed that P2D5-frozen RPE cells had the highest expression levels of RPE markers (BEST1, MITF, RLBP1, and PMEL17) of any time point studied (Fig. 2B). Immunohistochemistry revealed that expression of RLBP1 and ZO-1 (a tight junction marker) was significantly lower in P2D28-frozen RPE cells compared to the P2D5 and P2D11 groups (Fig. 2C). Furthermore, BEST1 and F-actin expression (stained with phalloidin) showed proper polarity in cells thawed from P2D5-frozen RPE, with BEST1 expressed on the basolateral side and F-actin on the apical side (Fig. 2D). However, in thawed cells from P2D11- and P2D28-frozen RPE, BEST1, and F-actin expression were reduced and mislocalized, indicating that polarity was impaired or even abolished in these groups of cells (Fig. 2D).

### Frozen RPE at P2D5 maintained function after thaw

**Fig. 3:**
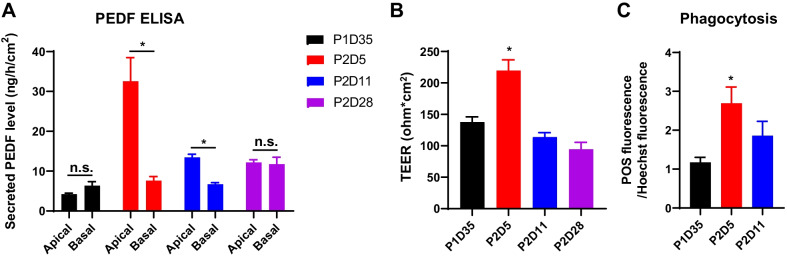
RPE frozen at P2D5 maintained the biological function after thawing. **A** Quantification analysis of PEDF secreted by thawed RPE cells frozen at indicated time points. PEDF secretion levels were measured by ELISA at D28 post thaw for both apical and basal compartments of transwell culture. **B** TEER of RPE cells at D28 post thaw. **C** Quantification analysis of relative fluorescence of 488-POS bound and ingested by RPE cells at D28 post thaw. *n* = 3 biological replicates. Data are represented as mean ± SD. Statistical differences are evaluated with two-way ANOVA with Bonferroni's post hoc test (**A**) or one-way ANOVA with Tukey's post hoc test (**B**, **C**). *P < 0.05

The biological function of thawed RPE cells was then evaluated by examining PEDF secretion, transepithelial electrical resistance (TEER), and phagocytosis ability at D28 post thaw. ELISA revealed that RPE cells thawed from P2D5 secreted a high level of PEDF into the apical medium, whereas the cells from the other groups secreted much lower levels of PEDF (Fig. 3A). There was no significant difference in apical and basal levels of PEDF between the P1D35 and P2D28 groups, confirming the loss of cell polarity (Fig. 3A). P2D5 cells had a significantly higher TEER after thaw compared to other time points (Fig. 3B). Furthermore, an Alexa Fluor 488-labeled porcine rod outer segments (POS) phagocytosis assay revealed that P2D5 RPE had the best phagocytic ability compared post thaw (Fig. 3C). Overall, these findings demonstrated that freezing RPE at P2D5 preserved optimal biological functions after thaw.

### P2D5 was the optimal time point for RPE cryopreservation regardless of freezing medium

To confirm that P2D5 was the best time for RPE cryopreservation, we used Genxin, a DMSO-free freezing medium, to cryopreserve the hESC-derived RPE cells. As expected, freezing RPE at P2D5 resulted in a higher attachment rate at D1 after thaw (Additional file 1: Fig. S1A). P2D5-frozen RPE cells expressed significantly higher levels of RPE markers at D28 post thaw than P2D11-frozen RPE cells (Additional file 1: Fig. S1B), indicating that freezing RPE at P2D5 provided the best post-thaw outcome regardless of the freezing medium used.

### Cell cycle and ECM-associated gene expression was enriched in P2D5 RPE

**Fig. 4:**
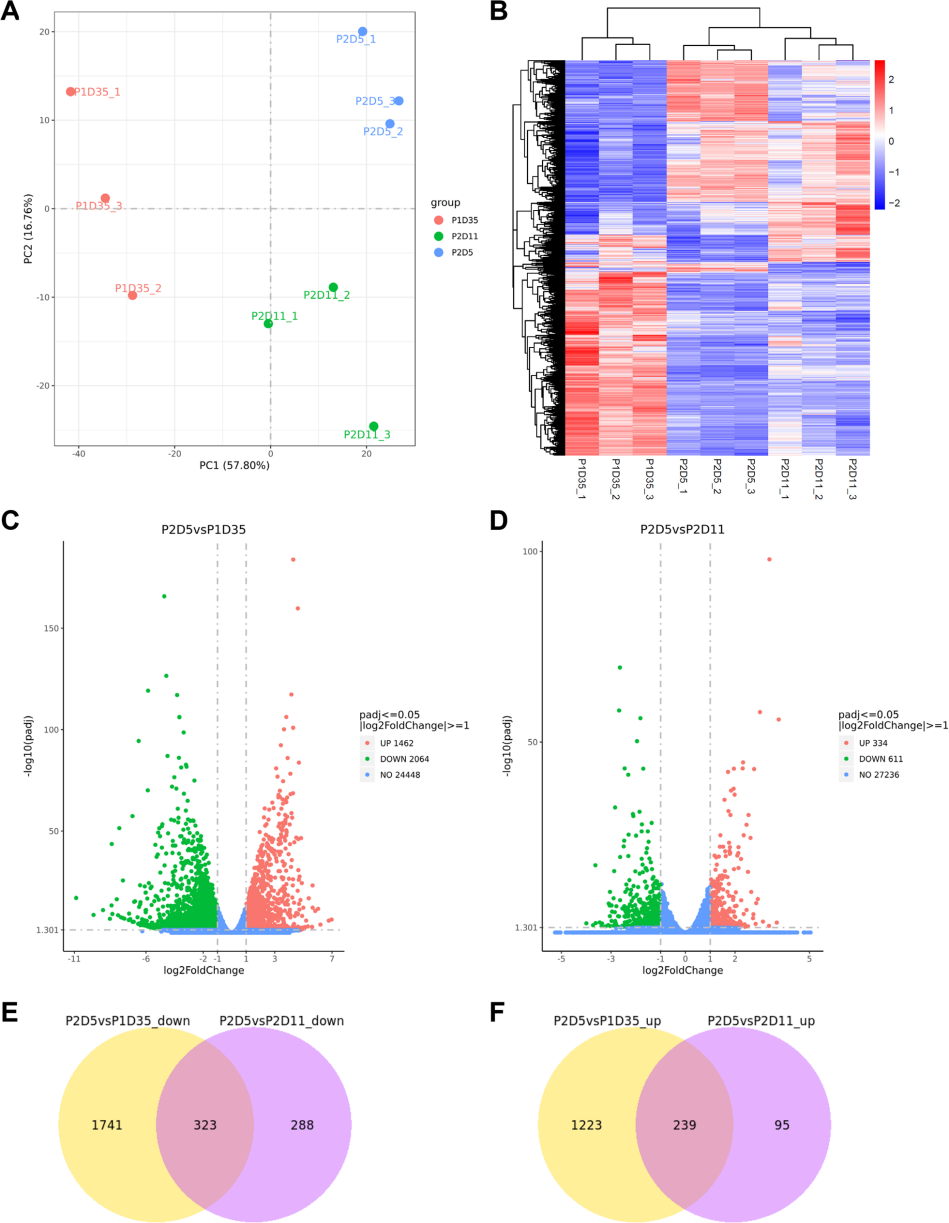
PCA and DEG analysis among the three groups of hESC-derived RPE. **A** PCA analysis of transcriptome for three-time points. B. Hierarchical clustering of all DEGs. **C**, **D**. Volcano plots of DEGs between P2D5 and P1D35 (P2D5 vs. P1D35) (**C**) and between P2D5 and P2D11 (**D**), respectively. Genes without significant differences are indicated by blue dots. The red dots indicate the upregulated genes, and the green dots indicate the down-regulated genes. Genes that are not significantly different between the two groups are indicated by blue dots.** E**. Venn diagram showing the overlap in the down-regulated DEGs between P2D5 versus P1D35 and P2D5 versus P2D11. **F** Venn diagram showing the overlap in the upregulated DEGs between P2D5 versus P1D35 and P2D5 versus P2D11. The numbers in each circle indicate the total number of different genes in each comparison group, and the number in the overlapping area represents the number of common genes between the two comparison groups

To investigate the molecular mechanisms underlying RPE cell tolerance to freezing at P2D5, we used RNA-seq to analyze RNA isolated from three independent cultures of hESC-derived RPE cells at P1D35, P2D5, and P2D11, respectively. The expected grouping among replicates of each time point was demonstrated by principal component analysis (PCA), with PC1 accounting for 57.8 percent of the variance and PC2 accounting for an additional 16.76% (Fig. 4A). We identified 4600 DEGs from RNA-seq data using the fold change > 2 and *P*_adj_ < 0.05 for comparisons between the three groups. Heatmap and hierarchical clustering analysis of all samples revealed distinct expression patterns of DEG sub-clusters and revealed that P2D5's transcriptomic profile resembled P2D11 more than P1D35 (Fig. 4B). 1462 DEGs were found to be upregulated in the P2D5 versus P1D35 comparison group, while 2064 were found to be down-regulated (Fig. 4C). 334 DEGs were found to be upregulated in the P2D5 vs. P2D11 comparison group, while 611 were found to be down-regulated (Fig. 4D). Venn diagrams revealed that when comparing P2D5 to the other two groups, 323 genes were down-regulated and 239 were upregulated (Fig. 4E, F).

**Fig. 5:**
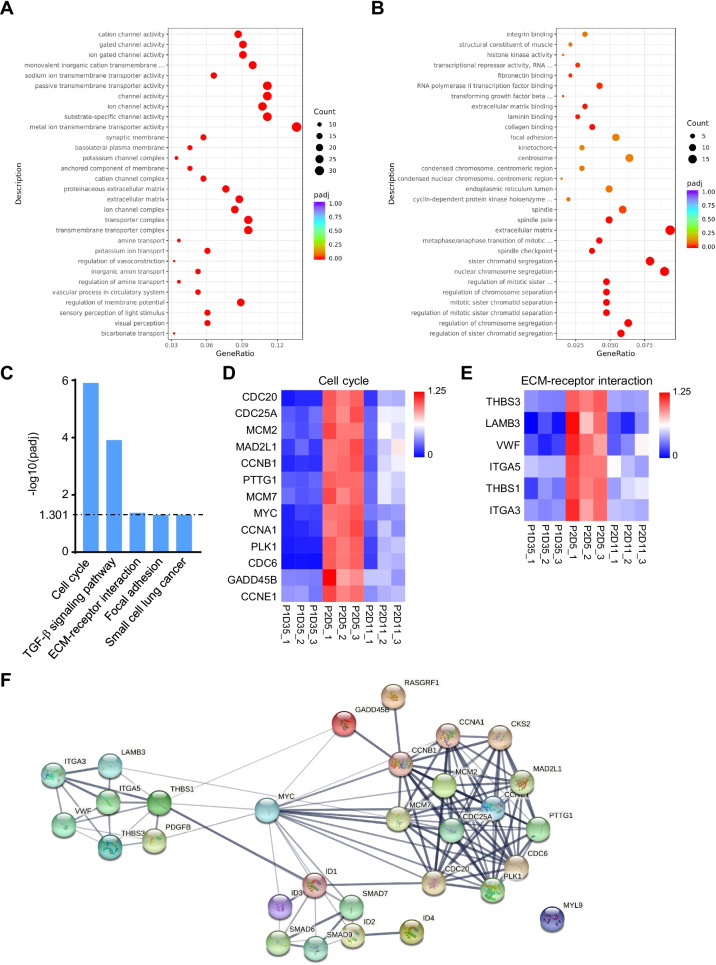
GO enrichment and KEGG pathway analysis. **A** Dot plots displaying the top 30 enriched GO categories among the shared down-regulated DEGs in P2D5 versus P1D35 and P2D11. **B** Dot plots displaying the top 30 enriched GO categories among the shared upregulated DEGs in P2D5 versus P1D35 and P2D11. The size of the circles indicates the number of DEGs that fall into the GO terms. The color of the circle represents the significance of the enriched GO terms. **C** KEGG pathways significantly enriched in shared upregulated DEGs in P2D5 vesus P1D35 and P2D11 (*P*_adj_ < 0.05). **D** Heatmap representing the relative FPKM of cell cycle-associated genes indicated by KEGG pathway analysis among the samples (normalized to average FPKM of P2D5 group). **E** Heatmap represented the FPKM of ECM-receptor interaction associated genes indicated by KEGG pathway analysis among the samples (normalized to average FPKM of P2D5 group). **F** Protein–protein interaction (PPI) network diagram of the DEGs that were clustered in significantly enriched KEGG pathways. Line thickness indicates the strength of data support

Because RPE cells at P2D5 were more resistant to freezing than P1D35 and P2D11, these shared DEGs that were either up or down-regulated in P2D5 vs. the other two groups are more likely to play roles in cell tolerance to freezing. According to Gene Ontology (GO) analysis, the shared down-regulated genes in P2D5 were enriched in visual perception, ion channel activity, and transmembrane transport activity, all of which were associated with RPE cell maturation, implying that RPE cells at P2D5 were indeed immature (Fig. 5A). Noteworthily, the shared upregulated genes in P2D5 were enriched in the GO of extracellular matrix (ECM) binding and chromosome segregation, an important event during cell mitosis (Fig. 5B). The KEGG pathway analysis revealed that the cell cycle, ECM-cell interaction, and focal-adhesion pathways were among the only five significantly enriched pathways (*P*_adj_ < 0.05). (Fig. 5C). Indeed, 13 cell cycle-related genes and 6 ECM-cell interaction related genes (overlapping with focal-adhesion related genes) were found to be highly expressed exclusively in RPE cells at P2D5 (Fig. 5D, E). The STRING database was used to analyze further the interactions of the clustered genes enriched from KEGG analysis, which revealed that THBS1 and MYC were located at the core of the interaction network diagram (Fig. 5F). Overall, these findings suggested that high expression of cell cycle/mitosis genes and ECM-associated genes may contribute to P2D5 RPE cell tolerance to freezing.

### hESC-derived RPE experienced a 1-week exponential stage upon passaging

**Fig. 6:**
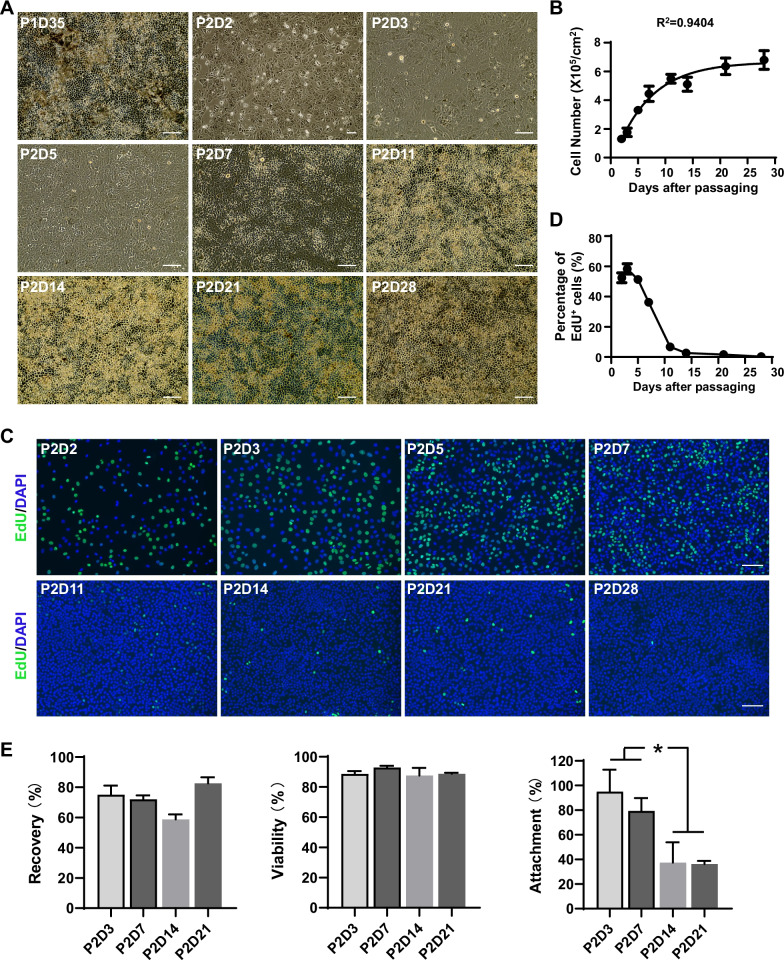
hESC-derived RPE required a 1-week exponential stage upon passaging. **A** Representative bright-field images showed the morphology of hESC-derived RPE cells at different time points in culture. **B** The growth curve of hESC-derived RPE was plotted as the cell number versus culture time. The data were fitted with an exponential-plateau model with *R*^2^ = 0.9404. **C** Representative fluorescent images of EdU-labeled RPE cells at different time points in culture. The nuclei were counter-stained with DAPI. Scale bars: 100 µm. **D** The quantification of the percentage of EdU-labeled RPE cells at different time points in culture. **E** The recovery, viability, and attachment rates were measured upon thawing of hESC-derived RPE cells frozen at indicated time points. *n* = 3 independent experiments; Data are represented as mean ± SD. Statistical differences are evaluated with one-way ANOVA with Tukey's post hoc test. *P < 0.05

To validate the RNA-seq analysis results, we observed the cell morphology and counted the cell number over time in hESC-derived RPE. After being passaged, the cells lost their hexagonal morphology and melanin pigmentation (Fig. 6A). After 10 days, the cells gradually reestablished the tight junction and regained hexagonal morphology and pigmentation. During the first week, RPE cells increased steadily and gradually. Following that, the growth rate slowed dramatically, and the cell number reached a plateau (Fig. 6B). We performed an EdU labeling assay over a 24-h period to further detect DNA synthesis in proliferating RPE cells [36]. EdU incorporation was abundant in the first week (Fig. 6C), indicating that the cells were actively proliferating. Only sporadic EdU incorporation was observed on days 11–14 (Fig. 6C, D). By day 28 (D28), the cells had matured, with increased melanin pigmentation and little EdU incorporation (Fig. 6A, C, D). Furthermore, four groups of RPE marker genes, including MITF, TYR and PMEL17 (pigmentation associated), RDH5, RLBP1 and RPE65 (visual cycle associated), BEST1, EZR and ZO-1 (polarity and tight junction), and VEGFA/B and PEDF (secretion), were selected from the RNA-seq data and their expression pattern indicated that RPE cells at P2D5 were immature and less functional compared to other time points (Additional file 1: Fig. S2). These findings demonstrated that the hESC-derived RPEs cells entered an exponential stage after passaging and underwent dedifferentiation only in the first week. As a result, RPE cells at P2D5 were proliferative with active DNA synthesis, consistent with the RNA-seq findings. To confirm that freezing RPE during the exponential phase is critical for cryopreservation success, we frozed cells at P2D3, P2D7, P2D14, and P2D21 and examined the recovery and survival, and attachment rates after thawing. Indeed, RPE cells frozen at P2D3 and P2D7 at the exponential phase showed significantly higher attachment rates than other time points (Fig. 6E), confirming that the exponential phase is optimal for RPE cryopreservation.

## Discussion

The clinical use of hESC-derived RPE in cell-replacement therapy for retinal degenerative diseases requires successful cryopreservation. However, it is unclear whether the freezing time point/phase affects the success of cryopreservation. To answer this question, we froze hESC-derived RPE cells at various time points (P1D35, P2D5, P2D11, and P2D28) after seeding and found that the cryopreserved cells at P2D5 retained the best RPE cellular phenotype and function in vitro and exhibited the highest post-thaw viability. Furthermore, RNA-seq analysis was performed and differentially expressed genes (DEGs) were identified in P2D5 RPE cells compared with other two groups (P1D35 and P2D11). Gene Ontology analysis was implemented to recognize the functions of DEGs in P2D5 group. KEGG pathway enrichment and PPI network were employed to address the key elements responsible for better cryopreservation outcomes.

The main objective of this study was to investigate the optimal timing for processing and cryopreservation of hESC-derived RPE cells. The first step was to analyze the recovery rate and viability rate for cells frozen at different timepoints (P1D35, P2D5, P2D11, and P2D28). Our results showed that in all cases, cryopreservation of RPE in CS10 maintained a high cell recovery and cell viability (Figs. 1, 6). No difference was observed between cells frozen at different timepoints right after thawing (Fig. 1, 6). For cells frozen at P2D11 and P2D28, their recovery rate was lower than viability rate, suggesting relatively more cells were lost during washing (Fig. 1). Further characterization was performed to monitor RPE molecular and phenotypic characteristics and function after cryopreservation. Molecular injuries could take hours or days to manifest. Herein, we evaluated apoptosis and cell attachment after 24-h culture. As shown in Fig. 1D, cell attachment of RPE frozen at different timepoints was variably affected. The attachment rate observed in cells frozen at P2D5 remained high (88%), while the percent of attached cells reduced to less than 35% for cells frozen at P1D35, P2D11, and P2D28 (33%, 32% and 35%, respectively). Of the detached cells in P2D5 group, about 8% were apoptotic, 10% in P1D35, 24% in P2D11, and 37% P2D28 groups (Fig. 1F), suggesting that lower adherence capability but not apoptosis was the key factor for cell loss. Functional assessment was performed after 28 days of culture. As a result, cryopreserved P2D5 cells exhibited a more homogeneous population with hexagonal shape, better epithelial polarity, better tight junctions that resulted in higher TEER values, RPE-related gene expression, PEDF secretion, and phagocytosis activity post-thaw compared with other groups (Figs. 2, 3). We noted patches of cells presented with darker pigmentation and a stronger RLBP1 immunostaining in the culture well of cells frozen at P2D28 in comparison with the other groups (Fig. 2A, C). This might be due to the longer culture time before freezing, which enhanced maturation of those cells. Unlike the P2D5 group, which grew into highly pure RPE cells, cells frozen at P2D28 grew into heterogeneous RPE population with more fibroblast-like cells after 28 days of culture post thaw. As a result, the average expression levels of pigmentation related genes (MITF and PMEL17) and RLBP1 were significantly lower than cells frozen at P2D5 (Fig. 2B). Overall, the choice of freezing time significantly affected the thawing outcomes and the RPE cells frozen at P2D5 recovered best after thaw.

RNA-Seq enabled us to compare gene profiles of RPE cells at different culture time points. As a result, a total of 562 DEGs, including 323 down-regulated and 239 upregulated DEGs, were identified in P2D5 cells. KEGG analysis of DEGs exclusively upregulated in P2D5 revealed that the DEGs were mainly enriched in five signal pathways, such as cell cycle, ECM-cell interaction, and focal-adhesion pathways and likely contributed to increased proliferation and adhesion of P2D5 cells (Figs. 4, 5). Cell cycle was the most enriched signal pathway for the P2D5-high DEGs (Fig. 5C). To validate the transcriptomic data on the proliferation state of cells in each group, cell growth curve analysis and EdU cell proliferation assay were performed. As shown in Fig. 6, P2D5 cells demonstrated a higher proliferation rate than the other 3 groups. They grew exponentially (in exponential phase) while P2D11 cells had entered a deceleration phase, and P2D28 had almost no proliferation. Previous studies have shown that induced pluripotent stem cells (iPSC) were also found to be best cryopreserved at the exponential growth phase [37]. We therefore hypothesized that exponential phase was the optimal growth phase for RPE cryopreservation and then addressed this hypothesis by comparison of the recovery rate, viability rate and attachment rate for cells frozen at different phases of the growth. Our results confirmed that cells at exponential growth phase (P2D3, P2D5 and P2D7) favored a good recovery (Fig. 1, 6). We noticed that the changes in growth rate were correlated with those in morphology. The characteristic hexagonal morphology and pigmentation were lost during the lag phase (around 2 days). RPE cells gradually reestablished hexagonal morphology in the exponential phase (around 5 days) and entered the deceleration phase (around 6 days) with more polygonal morphology (Fig. 6A, C, D). At this time, pigmentation levels were still low, although patches of pigmentation could be observed. Cells became polarized, hexagonal and fully pigmented at the stationary stage. Although the morphological changes of the RPE cells were useful to grossly evaluate stages of cells, hESC-RPE cells from the same well did not always mature with the same speed, which made accurate quantification challenging. It would be impractical to use the cell morphology as the selection criterion for timing of RPE cryopreservation. Previous studies have suggested that RPE cells freeze best prior to pigmentation, which was between 2 to 7 days after plating [19, 38]. For our cells, pigmentation started to show at around day 3 and freezing cells at day 3 did not perform better than other timepoints in exponential growth phase (Fig. 6E). Although the expression levels of pigmentation related genes in P2D5 group showed a tendency toward downregulation compared to the other groups, the differences did not reach statistical significance (Additional file 1: Fig. S2). This was not surprising to us since it had been reported earlier that the level of pigmentation did not reflect the maturation state of hESC-RPE [39]. However, we cannot rule out that pigment has some role in cryopreservation tolerance. The role of pigmentation in cryopreservation requires more investigation. Taken together, we propose that RPE cells at the exponential phase can be selected for cryopreservation. Since the cell state may vary between differentiation methods or cell lines, determination of the exponential phase is preferred for RPE cultured in different labs before freezing the cells.

To define the core factors responsible for the improved post-thaw outcomes of P2D5 group, GO (Gene Ontology) and STRING analysis were conducted. GO analysis identified that biological processes and molecular functions associated with RPE cell polarity and maturation were down-regulated while those associated with extracellular matrix (ECM) binding and cell cycles were significantly upregulated in the P2D5 group (Fig. 5A), indicating the less maturation, active proliferation, and higher attachment properties of P2D5 cells. These interconnected properties of P2D5 cells may be vital for cell recovery post thaw [40]. Our results corroborated previous reports that had suggested a correlation between the stage of differentiation and abilities to adhere and thrive post thaw [41] as well as a report on the cryopreservation of mesenchymal stem cells, where upregulation of ECM-related genes was correlated with the improved post-thaw function [42]. STRING database was used to conduct protein–protein interactions (PPI) network of the DEGs exclusively significantly enriched in five KEGG pathways of P2D5. MYC, Thrombospondin-1 (THBS1) and ID1, were identified as the hub genes of PPI. Considering the central role of ECM-cell interactions in cell adhesion and differentiation, the ECM molecule THBS1 drew our attention. RPE cells produce and secrete THBS1 [43], and it is intergrated into the laminin-rich extracellular matrix. THBS1 is recognized by multiple integrin receptors[44]. At P2D5, subunits of laminin, LAMB3, as well as the two integrin family members, ITGA3 and ITGA5, were found highly expressed (Fig. 5E). It is possible that THBS1 plays a key role in the formation of focal adhesions during attachment of cells [45, 46]. Studies have identified THBS1 partnering with different proteins to affect proliferation [44]. As an important regulator for ECM adhesion control and cell proliferation, THBS1 could be a potential target for improvement in RPE cryopreservation. However, more studies are needed to confirm this.

Aside from the cell cycle and ECM-associated pathways, KEGG analysis revealed that TGF-β signaling was more prevalent in P2D5 than P1D35 and P2D11. We observed that the cells displayed a fibroblastic-like morphology during the first week after passaging, particularly on day 5. Because TGF-β is known to be a potent inducer of EMT and pro-fibrosis in many organs, including the eyes, we speculate that the enriched TGF-β pathway in P2D5 RPE cells is linked to the transient EMT that occurs shortly after passage [47, 48]. Modulation of TGF-β pathway, either by adding TGF-β or by treatment with the TGF-β inhibitor SB431542, had no discernible effect on post-thaw attachment rate (data not shown), implying that the TGF-β pathway is not involved in cell tolerability to freezing.

Although this study focuses on determining the optimal stage for RPE cryopreservation, other critical factors, such as cooling rate and freezing solution, should be considered to achieve the best results. This study primarily used CryoStor CS10, a widely used cryopreservation medium containing 10% dimethylsulfoxide (DMSO) as a cryogenic protection agent (CPA). While CS10 is a cryopreservation solution designed specifically for human use, there are still concerns that DMSO may cause toxicity, impair normal cell function, and impede its use in cell-replacement therapy for retinal degenerative diseases. Our study also investigated the possibility of cryopreservation of RPE cells in another animal component-free cryopreservation solution (Genxin) that did not contain DMSO in this study (Additional file 1: Fig. S1). The results were preliminary but exciting, as the RPE at P2D5 frozen in Genxin demonstrated an equally high post-thaw attachment rate as that in CS10, indicating that CPA other than DMSO may be applicable for RPE cryopreservation and P2D5 is the optimal time point for RPE cryopreservation regardless of freezing medium.

## Limitations

This study was not designed to evaluate optimal freezing time of RPE derived from different stem cells lines using different protocols for differentiation. RPE derived from different stem cells lines using alternate methods may differ in maturity or expression of adhesion proteins, which could affect the cryopreservation process. A previous study reported that freezing the hiPSC-derived RPE cells at P1D14 (56 days after differentiation) could obtain good post-thaw functional recovery, when the thawed cells were passaged once and then cultured for 2–14 weeks [21]. It seems possible to freeze RPE cells at different passages. However, the time needed to regain a mature phenotype after thaw may vary. Likewise we did not investigate cryopreservation of adherent cells, although a recent study found that immature hESC-RPE adherent to a parylene scaffold is amenable to cryopreservation [19]. It is possible that cells exponential growth phase may recover from thaw even better if adhered to a scaffold. Another limitation of this study is that we only evaluated two different types of cryopreservation media. We used a commercial cell cryopreservation media CS10 containing DMSO and did a preliminary test using another commercial cell cryopreservation media Genxin containing no DMSO. Different cryopreservation media might prevent cryoinjury in different ways, thus variations in cryopreservation media may have an influence on which freezing time gives the best thawing outcomes. Effects of different cryopreservation media on optimal freezing time require further evaluation.

## Conclusions

Our studies show the cryopreservation of hESC-derived RPE cells at P2D5 produced the best results in terms of survival, attachment, and function after thaw. RPE cells at P2D5 were in the exponential phase with active DNA synthesis and expressed a high level of cell cycle/mitosis and ECM-associated genes, which we believe contributed to post-thaw survival. Our findings provide a new insight for successful cryopreservation of RPE cells for clinical application and might serve as a paradigm for studying the optimal stage for cryopreservation of other differentiated cell types.

## Supplementary Information

**Additional file 1.** Supplementary figures and table.

## Data Availability

The RNA-seq datasets (GSE197165) analyzed in this study are available in the Gene Expression Omnibus (GEO) database (http://www.ncbi.nih.gov/geo/). Other datasets are available from the corresponding author on reasonable request.

## Abbreviations

hESC: Human embryonic stem cells
RPE: Retinal pigment epithelium
EMT: Epithelial-to-mesenchymal transition
EdU: 5‐Ethynyl‐2′‐deoxyuridine
PEDF: Pigment epithelium-derived factor
OS: Photoreceptor outer segments
POS: Porcine rod outer segments
TEER: Transepithelial electrical resistance
ELISA: Enzyme-linked immunosorbent assay
RT-qPCR: Reverse transcription-quantitative PCR
RNA-seq: Ribonucleic acid sequencing
DEGs: Differentially expressed genes
GO: Gene ontology
KEGG: Kyoto encyclopedia of genes and genomes
PPI: Protein–protein interaction
ECM: Extracellular matrix
THBS1: Thrombospondin-1
PFA: Paraformaldehyde
FBS: Fetal bovine serum
BSA: Bovine serum albumin
PBS: Phosphate-buffered saline

## References

[1] Lakkaraju A, Umapathy A, Tan LX, Daniele L, Philp NJ, Boesze-Battaglia K, Williams DS. The cell biology of the retinal pigment epithelium. Prog Retin Eye Res. 2020;78:100846. 10.1016/j.preteyeres.2020.100846 PMC8941496 32105772

[2] McBain VA, Townend J, Lois N (2012). Progression of retinal pigment epithelial atrophy in stargardt disease. Am J Ophthalmol.

[3] Pan CK, Heilweil G, Lanza R, Schwartz SD (2013). Embryonic stem cells as a treatment for macular degeneration. Expert Opin Biol Ther.

[4] Bertolotti E, Neri A, Camparini M, Macaluso C, Marigo V (2014). Stem cells as source for retinal pigment epithelium transplantation. Prog Retin Eye Res.

[5] Mehat MS, Sundaram V, Ripamonti C, Robson AG, Smith AJ, Borooah S, Robinson M, Rosenthal AN, Innes W, Weleber RG (2018). Transplantation of human embryonic stem cell-derived retinal pigment epithelial cells in macular degeneration. Ophthalmology.

[6] da Cruz L, Fynes K, Georgiadis O, Kerby J, Luo YH, Ahmado A, Vernon A, Daniels JT, Nommiste B, Hasan SM (2018). Phase 1 clinical study of an embryonic stem cell-derived retinal pigment epithelium patch in age-related macular degeneration. Nat Biotechnol.

[7] Kashani AH, Lebkowski JS, Rahhal FM, Avery RL, Salehi-Had H, Dang W, Lin CM, Mitra D, Zhu D, Thomas BB, et al. A bioengineered retinal pigment epithelial monolayer for advanced, dry age-related macular degeneration. Sci Transl Med. 2018;10(435):eaao4097. 10.1126/scitranslmed.aao4097 29618560

[8] Kashani AH, Lebkowski JS, Rahhal FM, Avery RL, Salehi-Had H, Chen S, Chan C, Palejwala N, Ingram A, Dang W (2021). One-year follow-up in a phase 1/2a clinical trial of an allogeneic RPE cell bioengineered implant for advanced dry age-related macular degeneration. Transl Vis Sci Technol.

[9] Wang Y, Zhang D, Shen B, Zhang Y, Gu P (2018). Stem/progenitor cells and biodegradable scaffolds in the treatment of retinal degenerative diseases. Curr Stem Cell Res Ther.

[10] Hunt NC, Hallam D, Chichagova V, Steel DH, Lako M (2018). The Application of biomaterials to tissue engineering neural retina and retinal pigment epithelium. Adv Healthc Mater.

[11] Leach LL, Clegg DO (2015). Concise review: making stem cells retinal: methods for deriving retinal pigment epithelium and implications for patients with ocular disease. Stem Cells.

[12] Maeda T, Mandai M, Sugita S, Kime C, Takahashi M. Strategies of pluripotent stem cell-based therapy for retinal degeneration: update and challenges. Trends Mol Med. 2022;28(5):388–404. 10.1016/j.molmed.2022.03.001 35370091

[13] Rizzolo LJ, Nasonkin IO, Adelman RA (2022). Retinal cell transplantation, biomaterials, and in vitro models for developing next-generation therapies of age-related macular degeneration. Stem Cells Transl Med.

[14] Woods EJ, Thirumala S, Badhe-Buchanan SS, Clarke D, Mathew AJ (2016). Off the shelf cellular therapeutics: factors to consider during cryopreservation and storage of human cells for clinical use. Cytotherapy.

[15] Kitahata S, Tanaka Y, Hori K, Kime C, Sugita S, Ueda H, Takahashi M (2019). Critical functionality effects from storage temperature on human induced pluripotent stem cell-derived retinal pigment epithelium cell suspensions. Sci Rep.

[16] Pasovic L, Utheim TP, Reppe S, Khan AZ, Jackson CJ, Thiede B, Berg JP, Messelt EB, Eidet JR (2018). Improvement of storage medium for cultured human retinal pigment epithelial cells using factorial design. Sci Rep.

[17] Pasovic L, Eidet JR, Lyberg T, Messelt EB, Aabel P, Utheim TP (2014). Antioxidants improve the viability of stored adult retinal pigment epithelial-19 cultures. Ophthalmol Ther.

[18] Buchholz DE, Pennington BO, Croze RH, Hinman CR, Coffey PJ, Clegg DO (2013). Rapid and efficient directed differentiation of human pluripotent stem cells into retinal pigmented epithelium. Stem Cells Transl Med.

[19] Pennington BO, Bailey JK, Faynus MA, Hinman C, Hee MN, Ritts R, Nadar V, Zhu D, Mitra D, Martinez-Camarillo JC (2021). Xeno-free cryopreservation of adherent retinal pigmented epithelium yields viable and functional cells in vitro and in vivo. Sci Rep.

[20] Brandl C, Zimmermann SJ, Milenkovic VM, Rosendahl SM, Grassmann F, Milenkovic A, Hehr U, Federlin M, Wetzel CH, Helbig H (2014). In-depth characterisation of Retinal Pigment Epithelium (RPE) cells derived from human induced pluripotent stem cells (hiPSC). Neuromol Med.

[21] Reichman S, Slembrouck A, Gagliardi G, Chaffiol A, Terray A, Nanteau C, Potey A, Belle M, Rabesandratana O, Duebel J (2017). Generation of storable retinal organoids and retinal pigmented epithelium from adherent human ips cells in xeno-free and feeder-free conditions. Stem Cells.

[22] Li QY, Zou T, Gong Y, Chen SY, Zeng YX, Gao LX, Weng CH, Xu HW, Yin ZQ (2021). Functional assessment of cryopreserved clinical grade hESC-RPE cells as a qualified cell source for stem cell therapy of retinal degenerative diseases. Exp Eye Res.

[23] Hongisto H, Ilmarinen T, Vattulainen M, Mikhailova A, Skottman H (2017). Xeno- and feeder-free differentiation of human pluripotent stem cells to two distinct ocular epithelial cell types using simple modifications of one method. Stem Cell Res Ther.

[24] Maminishkis A, Chen S, Jalickee S, Banzon T, Shi G, Wang FE, Ehalt T, Hammer JA, Miller SS (2006). Confluent monolayers of cultured human fetal retinal pigment epithelium exhibit morphology and physiology of native tissue. Invest Ophthalmol Vis Sci.

[25] Jiang M, Esteve-Rudd J, Lopes VS, Diemer T, Lillo C, Rump A, Williams DS (2015). Microtubule motors transport phagosomes in the RPE, and lack of KLC1 leads to AMD-like pathogenesis. J Cell Biol.

[26] Parinot C, Rieu Q, Chatagnon J, Finnemann SC, Nandrot EF. Large-scale purification of porcine or bovine photoreceptor outer segments for phagocytosis assays on retinal pigment epithelial cells. J Vis Exp. 2014;(94):52100. 10.3791/52100 PMC4396958 25548986

[27] Mao Y, Finnemann SC (2013). Analysis of photoreceptor outer segment phagocytosis by RPE cells in culture. Methods Mol Biol.

[28] Kim D, Pertea G, Trapnell C, Pimentel H, Kelley R, Salzberg SL (2013). TopHat2: accurate alignment of transcriptomes in the presence of insertions, deletions and gene fusions. Genome Biol.

[29] Liao Y, Smyth GK, Shi W (2014). featureCounts: an efficient general purpose program for assigning sequence reads to genomic features. Bioinformatics.

[30] Wang L, Feng Z, Wang X, Wang X, Zhang X (2010). DEGseq: an R package for identifying differentially expressed genes from RNA-seq data. Bioinformatics.

[31] Saldanha AJ (2004). Java Treeview–extensible visualization of microarray data. Bioinformatics.

[32] Yu G, Wang LG, Han Y, He QY (2012). clusterProfiler: an R package for comparing biological themes among gene clusters. OMICS.

[33] Kanehisa M, Sato Y, Kawashima M, Furumichi M, Tanabe M (2016). KEGG as a reference resource for gene and protein annotation. Nucleic Acids Res.

[34] Szklarczyk D, Franceschini A, Kuhn M, Simonovic M, Roth A, Minguez P, Doerks T, Stark M, Muller J, Bork P (2011). The STRING database in 2011: functional interaction networks of proteins, globally integrated and scored. Nucleic Acids Res.

[35] Choudhary P, Booth H, Gutteridge A, Surmacz B, Louca I, Steer J, Kerby J, Whiting PJ (2017). Directing differentiation of pluripotent stem cells toward retinal pigment epithelium lineage. Stem Cells Transl Med.

[36] Chehrehasa F, Meedeniya AC, Dwyer P, Abrahamsen G, Mackay-Sim A (2009). EdU, a new thymidine analogue for labelling proliferating cells in the nervous system. J Neurosci Methods.

[37] Uhrig M, Ezquer F, Ezquer M. Improving cell recovery: freezing and thawing optimization of induced pluripotent stem cells. Cells. 2022;11(5):799–817. 10.3390/cells11050799 PMC8909336 35269421

[38] Leach LL, Croze RH, Hu Q, Nadar VP, Clevenger TN, Pennington BO, Gamm DM, Clegg DO (2016). Induced pluripotent stem cell-derived retinal pigmented epithelium: a comparative study between cell lines and differentiation methods. J Ocul Pharmacol Ther.

[39] Bennis A, Jacobs JG, Catsburg LAE, Ten Brink JB, Koster C, Schlingemann RO, van Meurs J, Gorgels T, Moerland PD, Heine VM (2017). Stem cell derived retinal pigment epithelium: the role of pigmentation as maturation marker and gene expression profile comparison with human endogenous retinal pigment epithelium. Stem Cell Rev Rep.

[40] Nicolas J, Magli S, Rabbachin L, Sampaolesi S, Nicotra F, Russo L (2020). 3D extracellular matrix mimics: fundamental concepts and role of materials chemistry to influence stem cell fate. Biomacromol.

[41] Schwartz SD, Hubschman JP, Heilwell G, Franco-Cardenas V, Pan CK, Ostrick RM, Mickunas E, Gay R, Klimanskaya I, Lanza R (2012). Embryonic stem cell trials for macular degeneration: a preliminary report. Lancet.

[42] Pollock K, Samsonraj RM, Dudakovic A, Thaler R, Stumbras A, McKenna DH, Dosa PI, van Wijnen AJ, Hubel A (2017). Improved post-thaw function and epigenetic changes in mesenchymal stromal cells cryopreserved using multicomponent osmolyte solutions. Stem Cells Dev.

[43] Miyajima-Uchida H, Hayashi H, Beppu R, Kuroki M, Fukami M, Arakawa F, Tomita Y, Kuroki M, Oshima K (2000). Production and accumulation of thrombospondin-1 in human retinal pigment epithelial cells. Invest Ophthalmol Vis Sci.

[44] Resovi A, Pinessi D, Chiorino G, Taraboletti G (2014). Current understanding of the thrombospondin-1 interactome. Matrix Biol.

[45] Bachmann M, Kukkurainen S, Hytonen VP, Wehrle-Haller B (2019). Cell adhesion by integrins. Physiol Rev.

[46] Kechagia JZ, Ivaska J, Roca-Cusachs P (2019). Integrins as biomechanical sensors of the microenvironment. Nat Rev Mol Cell Biol.

[47] Xu J, Lamouille S, Derynck R (2009). TGF-beta-induced epithelial to mesenchymal transition. Cell Res.

[48] Meng XM, Nikolic-Paterson DJ, Lan HY (2016). TGF-beta: the master regulator of fibrosis. Nat Rev Nephrol.

