# Supplementary material for: Determining the optimal stage for cryopreservation of human embryonic stem cell-derived retinal pigment epithelial cells
Source: Stem Cell Res Ther. 2022 Sep 5;13:454. doi: 10.1186/s13287-022-03141-2 (PMC9446586; doi:10.1186/s13287-022-03141-2)
Supplement: Supplementary file 1 — Additional file 1. Supplementary figures and table. [file 13287_2022_3141_MOESM1_ESM.docx]

**SUPPLEMENTARY DATA**

**Determining the optimal stage for cryopreservation of human embryonic stem cell-derived retinal pigment epithelial cells**

Ting Zhang, Xianyu Huang, Sujun Liu, Xinyue Bai, Xinyue Zhu, Dennis O. Clegg, Mei Jiang, Xiaodong Sun.

Supplementary data include 2 Supplementary figures and 1 Supplementary table.

**Supplementary Figure S1.** **P2D5 was the optimal time point for RPE cryopreservation regardless of freezing medium.**


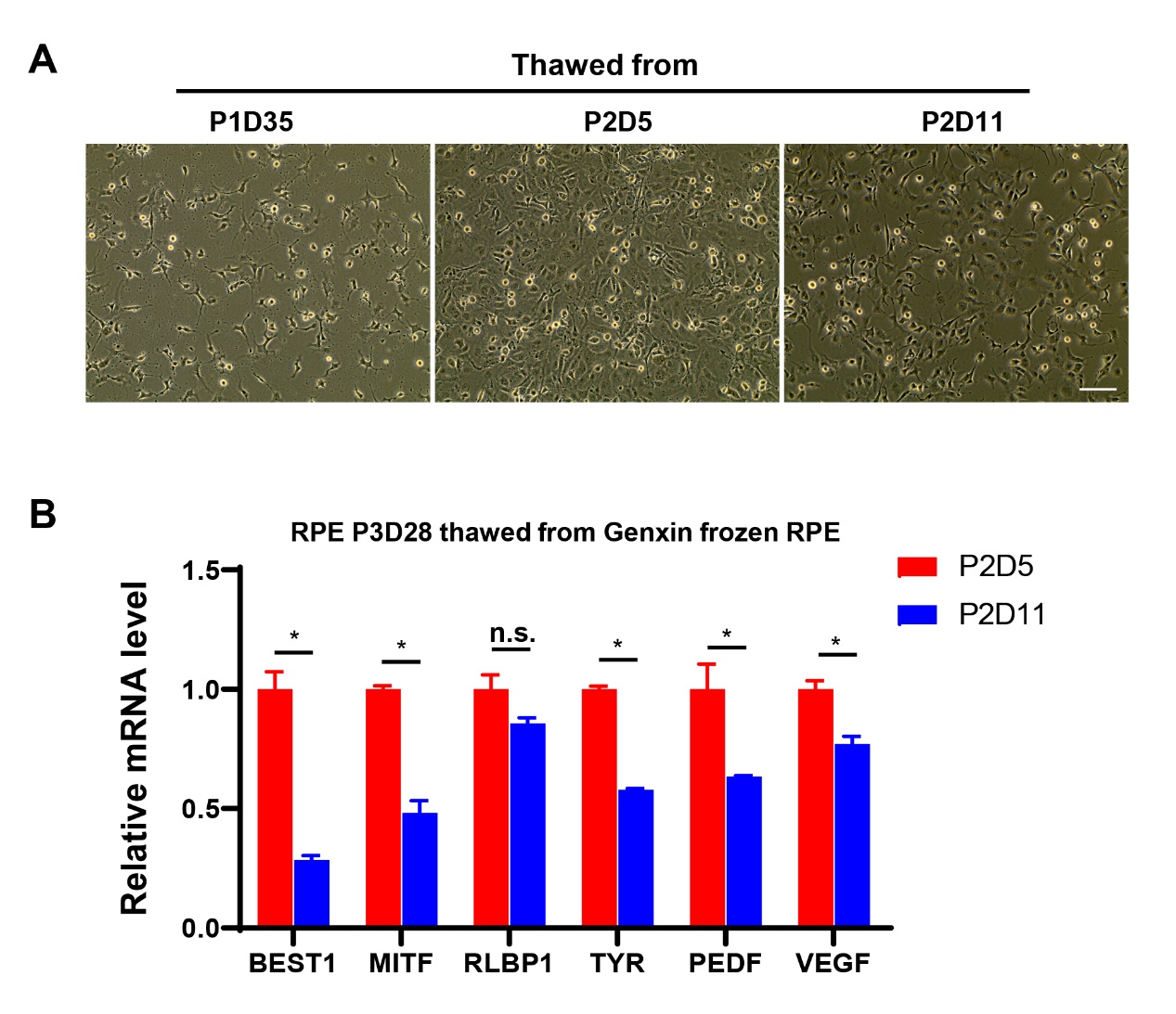


RPE cells were frozen in another freezing solution Genxin at different time points and the thawed cells were examined. A. The bright-field images of RPE cells from different groups 24 h post thaw, respectively. Scale bars: 100 μm. B. RT-qPCR analysis of relative mRNA expression levels of RPE marker genes BEST1, MITF, RLBP1, TYR, PEDF and VEGF at D28 post thaw. GAPDH was used as internal control, and the values were normalized to the P2D5 group for each marker gene. Data are represented as mean ± SD. Statistical differences are evaluated with two-way ANOVA with Bonferroni's post hoc test. **P* < 0.05. n.s. not significantly different.

**Supplementary Figure S2. P2D5 RPE cells were immature and less functional compared to other time points.**


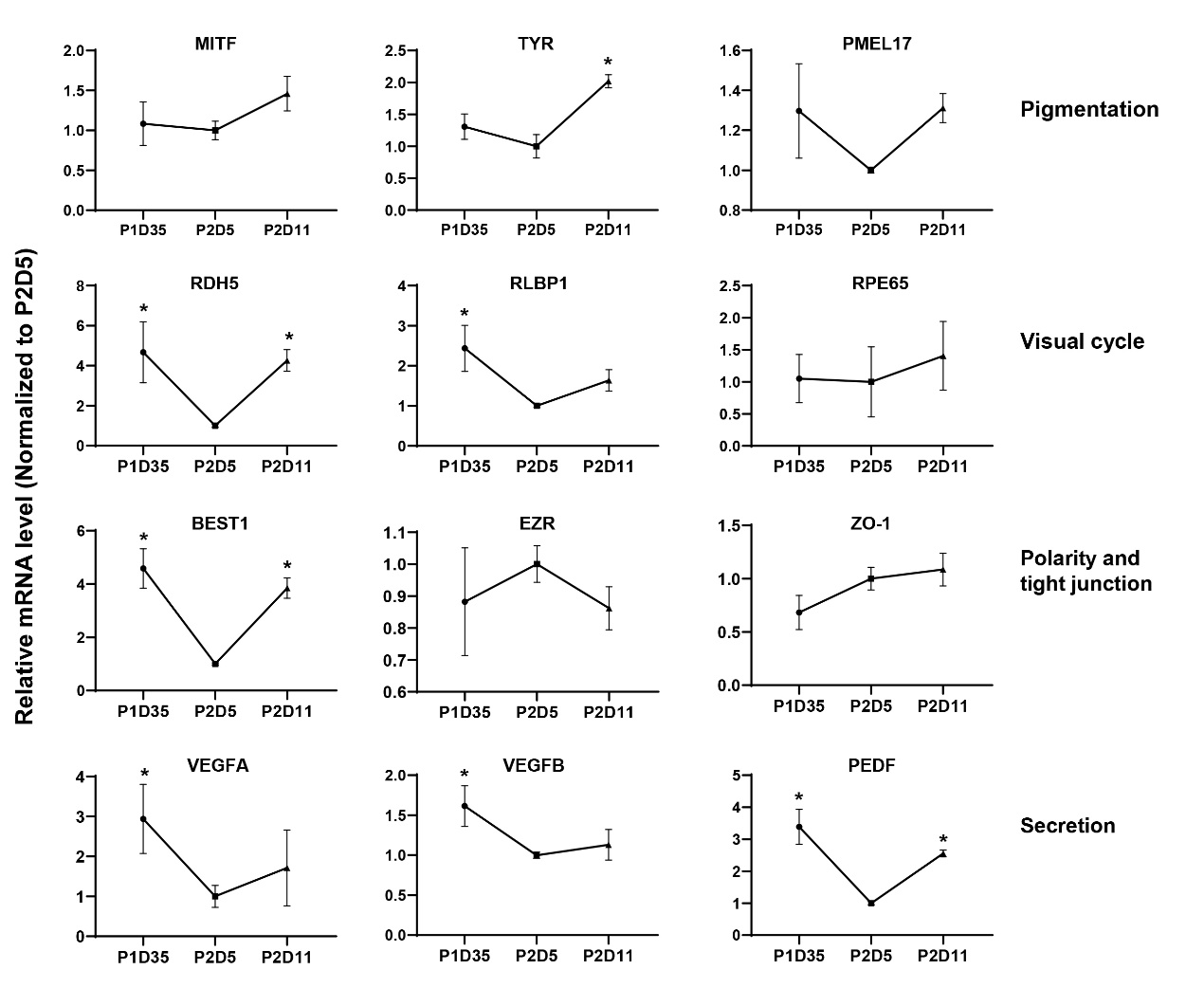


Four groups of RPE marker genes were selected from the RNA-seq data and their expression patterns were presented, respectively. The values of the relative mRNA levels were normalized to the P2D5 group for each marker gene. Data are represented as mean ± SD. Statistical differences are evaluated with one-way ANOVA with Tukey’s post hoc test. *P < 0.05.

**Supplementary Table S1.** Sequences of primers used for qPCR.

| Gene | Forward primer sequence (5’ to 3’) | Reverse primer sequence (5’ to 3’) |
| --- | --- | --- |
| GAPDH | GAGCACAAGAGGAAGAGAGAGACCC | GTTGAGCACAGGGTACTTTATTGATGGTACATG |
| MITF | ACTTTCACTCTTCGCCAAGG | TGCGTGATGTCATACTGGAG |
| BEST1 | CTGGGCTTCTACGTGACGC | TTGCTCGTCCTTGCCTTCG |
| RLBP1 | AAGCTGGCTACCCTGGTGT | TGAAGCAATATGCCTGCAAGA |
| PMEL17 | AGGTGCCTTTCTCCGTGAG | AGCTTCAGCCAGATAGCCACT |
| TYRO | CCATTGGACATAACCGGGAA | AAGGAGCCATGACCAGATCC |
| PEDF | TTCAAAGTCCCCGTGAACAAG | GAGAGCCCGGTGAATGATGG |
| VEGF | AGGGCAGAATCATCACGAAGT | AGGGTCTCGATTGGATGGCA |
